# Change Process in Coaching: Interplay of Nonverbal Synchrony, Working Alliance, Self-Regulation, and Goal Attainment

**DOI:** 10.3389/fpsyg.2021.580351

**Published:** 2021-06-23

**Authors:** Tünde Erdös, Fabian T. Ramseyer

**Affiliations:** ^1^Department of Management and Organization, Amsterdam Business Research Institute, Vrije Universiteit, Amsterdam, Netherlands; ^2^Department of Clinical Psychology and Psychotherapy, Institute of Psychology, University of Bern, Bern, Switzerland

**Keywords:** change process, nonverbal synchrony, goal attainment, coaching, self-regulation

## Abstract

**Background:** Psychological literature emphasizes that self-regulation is important as goal intentions, goal setting, or implementation intention does not automatically result in effective results in coaching. The question which coaching strategies to apply to strengthening clients' self-regulatory capacities as prerequisites of effective change outcomes remains a black box in coaching.

**Method:** This quantitative study explored clients' self-regulatory mechanisms by addressing how nonverbal synchrony influences clients' cognitive and emotional self-regulation across sessions. One hundred eighty-four coach–client pairs and their evolving change process were observed over 8 months. Video-recorded sessions were assessed with motion energy analysis to automatically capture coach and client nonverbal behavior and quantify nonverbal synchrony at the level of the dyad.

**Results:** Synchrony was differentially associated with clients' post-session questionnaires on result-oriented problem-reflection and self-reflection, affect balance, and working alliance. Network analyses suggested that the association between synchrony and other process variables did not correspond to the previously found positive association between synchrony and positive aspects of alliance or outcome. Instead, this association depended on the level of perceived outcome.

**Discussion:** Coaching success may be predicted by process variables assessed after each session: goal reflection, alliance, and mood all predict successful coaching. The assessment of nonverbal synchrony suggests a state-dependent effect of embodied processes on a coaching outcome that warrants further inspection.

## Introduction

Based on meta-analytic evidence, one may state that coaching works (De Meuse et al., 2009; Sonesh et al., 2015; Jones et al., 2016; Burt and Talati, 2017; Athanasopoulou and Dopson, 2018). Coaching has been described as an effective change methodology for clients (Grant et al., 2010), and it has been defined as a “*result-oriented, systematic process*” (Grant, 2003, p. 254), which could be simplified to be regarded as a goal-focused activity (Gregory et al., 2011). Despite such straightforward descriptions, changes of coaching effectiveness over the course of the coaching engagement remain elusive (Molyn et al., 2019), and thus the question of when and why coaches should apply which coaching strategies remains a black box (Theeboom et al., 2017).

The present study seeks to tap into this black box by exploring specific interactional processes through which effective change can be attained “*within and across coaching sessions, including the development of the relationship*” (Myers, 2017, p. 590). The coach-client relationship is perceived as the “*most important success factor in the coaching process*” (Gessnitzer and Kauffeld, 2015, p. 178). Thus, we are interested in how the quality of this relationship, which is commonly referred to as “working alliance” (Bordin, 1979) affects clients' self-regulation over the course of coaching. In sports coaching, the interplay between the coach-athlete relationship and self-regulation has been repeatedly reported (Jowett, 2017; Collins et al., 2018), and our recent literature review (Erdoes et al., 2020) indicated that change outcomes are driven by two main aspects: a client's cognitive and emotional processes.

Our focus regarding the coaching process is generally based on interpersonal theories (Kiesler, 1996; Horowitz, 2004) and on interpersonal adaptation at a more specific level (Bernieri and Rosenthal, 1991; Burgoon et al., 1995). Employing the framework of embodied cognition (e.g., Wachsmuth et al., 2008; Tschacher and Bergomi, 2011), we will focus on a coach's and a client's nonverbal behavior (Ramseyer and Tschacher, 2006) visible in video-recorded coaching sessions. For this aim, we introduce nonverbal synchrony—a measure of movement coordination between interaction partners—into the coaching literature (Erdoes, 2019).

Our project extends previous coaching studies that have included the nonverbal channel by looking at the degree of dominance and affiliation in coach–client interactions (Schermuly et al., 2010; Ianiro et al., 2013, 2015; Ianiro and Kauffeld, 2014). Specifically, we are interested in exploring the interplay between nonverbal synchrony, emotional as well as cognitive self-regulation, and their association with goal attainment in coaching.

## Conceptual Background

### Working Alliance

The working alliance as a therapeutic concept (Wampold and Imel, 2015) is characterized by a shared goal/task focus, trust, and rapport (Bordin, 1979). Numerous findings in psychotherapy research show that working alliance is the best-researched predictor for therapeutic outcomes (Flückiger et al., 2018). It has also been found to be of central importance to coaching effectiveness both in coaching process research (Gessnitzer and Kauffeld, 2015) and coaching outcome research (e.g., de Haan et al., 2013, 2016, 2019, 2020). Llewelyn and Hardy (2001) argued that there are sufficient similarities between psychotherapy and coaching for the literature on therapeutic process research to be considered in coaching (e.g., Peltier, 2011). Thus, working alliance has been transferred and adapted to coaching process research. For the purposes of the present study, we posit that working alliance plays a role in how nonverbal synchrony is associated with a client's goal attainment.

### Nonverbal Synchrony

Nonverbal synchrony refers to the coordination of (mostly) observable nonverbal phenomena between two or more interaction patners (Tschacher and Ramseyer, 2017). This aspect of interpersonal adaptation (Burgoon et al., 1995) has recently received increased attention in the domain of psychotherapy (Wiltshire et al., 2020) and social psychology (Mogan et al., 2017), but it has remained far less explored in coaching. While recent advances have been made in the domain of relating nonverbal synchrony to qualitative aspects of nonverbal behavior (Fujiwara et al., 2020; Altmann et al., 2021a,b), our conceptualization of nonverbal synchrony will be focused on movement *dynamics*, irrespective of movement quality or direction (Ramseyer, 2020b). Generally speaking, studies on synchrony in interpersonal relationships have increased in recent years (Chetouani et al., 2017), and numerous positive aspects of interactional synchrony and interpersonal relationships have been reported (Chartrand and Lakin, 2013), but nonverbal synchrony between a coach and a client as specified in this paper has, so far, received no attention in coaching psychology. This is unfortunate as phenomena of social coordination are both the product of and contribute to positive interactions (Stel and Vonk, 2010; Koole and Tschacher, 2016; Omer et al., 2019). More specifically, synchrony has been related to better joint performance (Cui et al., 2012), effective communication (Jiang et al., 2014), rapport (Bernieri et al., 1994; Hove and Risen, 2009; Miles et al., 2009), psychotherapy outcome (Ramseyer and Tschacher, 2011; Altmann et al., 2020; Cohen et al., 2020); empathy (Bavelas et al., 1986), the smoothness of conversation (Chartrand and Bargh, 1999), and to social connectedness in general (Marsh et al., 2009). Conclusively, in this study, we explicitly focus on synchronized whole-body movement of both a coach and a client in coaching sessions.

### Automated Measurement of Nonverbal Synchrony

One of the aims of the present study is to respond to the commonly shared limitation of low inter-rater reliability (Baesler and Burgoon, 1987) in general and the use of self-reports in coaching process research (Bozer et al., 2013). Therefore, we used a video-based analysis to study the body movement of both a coach and a client and to operationalize these objective measures to define what we call “coach–client nonverbal synchrony.” While there is a growing number of studies in research fields such as psychotherapy and developmental sciences investigating nonverbal synchrony, using automated measurements (Wiltshire et al., 2020), coaching research has remained focused on looking into effects of verbal (e.g., Cilliers, 2005; Schermuly and Scholl, 2012; Bachkirova et al., 2015; Gessnitzer and Kauffeld, 2015) rather than nonverbal behavior in coaching process research. To date, the few coaching studies on both verbal and nonverbal behavior (Ianiro et al., 2013, 2015; Ianiro and Kauffeld, 2014) have either focused on the nonverbal behavior of the client or that of the coach, showing, for instance, that the coach's nonverbal behavior plays a decisive role in the development of the coach–client relationship (Ianiro and Kauffeld, 2014). In the present article, we seek to extend these findings with the addition of nonverbal synchrony.

### Self-Regulation

We consider self-regulation to be a meta-cognitive monitoring ability (Greif and Berg, 2011) that focuses on result-oriented self-reflection rather than aimless rumination (Greif, 2008) and also affects emotion regulation (Hayes and Feldman, 2004; Feldman, 2015). Self-regulation integrates self-regulatory processes “*as the set of psychological processes through which people bring their thoughts, feelings, and behaviors in line with abstract standards, goals, or values*” (Koole et al., 2006, p. 206). These psychological processes amplify, attenuate, or maintain the strength of various emotional reactions (Gross and John, 1998; Davidson, 2000). Self-regulatory processes have been shown to reduce experiential avoidance (Hayes et al., 1996), thought suppression (Wegner, 1994), or over-engagement in worry (Borkovec, 1994), rumination (Nolen-Hoeksema and Morrow, 1991), and overgeneralization (Carver, 1998)—aspects that together facilitate emotional self-regulation (Kumar, 2002).

### Self-Regulation and Nonverbal Synchrony

Links between synchrony and emotional self-regulation have been reported in developmental research, where the synchronous interaction between an infant and a caregiver was revealed to be essential for the development of skills for emotional self-regulation in adolescence (Feldman, 2015). In particular, affect balance characterized by emotional safety was shown to foster self-control abilities in toddlers (Feldman et al., 1999). In a similar vein, mutual affect synchrony has been found to be associated with downregulating emotional distress (Feldman, 2007), and fostering emotional safety (Feldman, 2015). Comparable links between interpersonal synchrony and emotional self-regulation have been found in close relationships of adults (Butler and Randall, 2013; Ferrer and Helm, 2013; Timmons et al., 2015) where co-regulation was also seen as a form of healthy equilibrium of emotional responses (Reed et al., 2015). Co-regulation may also be found in physiological synchrony (Marci and Orr, 2006; Kleinbub, 2017).

Up to now, the link between nonverbal synchrony and emotion has been examined in student conversations (Tschacher et al., 2014), where synchrony in body movements predicted positive affect. We thus conceptualize self-regulation as the client's capacity to reflect goals and problems in a result-oriented manner (Watson et al., 1988; Greif and Berg, 2011). We, therefore, hypothesized as follows:

*Hypothesis 1a:*

In coaching, nonverbal synchrony (spontaneous movement coordination) increases a client's self-regulation capacities as operationalized through self-reported mood.

*Hypothesis 1b:*

In coaching, nonverbal synchrony increases a client's self-regulation capacities as operationalized through a result-oriented problem and self-reflection.

### Goal Attainment, Self-Regulation, and Nonverbal Synchrony

In coaching, effective goal attainment (Prywes, 2012) has been demonstrated to comprise cognitive processes such as goal-oriented planning (Wood and Locke, 1990), goal commitment (Locke and Latham, 1990), perceived goal competence (Sheldon et al., 1996), goal self-concordance (Sheldon and Houser-Marko, 2001), and goal stability (Spence et al., 2008). The way clients engage in effective goal-attainment in association with cognitive self-regulatory capacities has been recently demonstrated in sports coaching (Collins et al., 2018). Furthermore, conscientiousness (Costa and McCrae, 1992), as a specific personality characteristic, was found to consistently predict performance (Stewart et al., 2008). In the present study, we investigated these ingredients of goal attainment (Prywes, 2012) as direct effects of sustained goal-directed behavior. By assessing goals 3 months after completion of coaching, the present study sought to explore goal attainment as sustained goal-directed behavior through coaching. In clients where these ingredients of goal attainment are maintained after coaching, we understand the coaching engagement to have been successfully completed. In coaching, the highest quality form of goal attainment is attained when clients' “need to be autonomous” is met (Schiemann et al., 2018), when they attain goals through engagement in sustained goal-directed behavior beyond coaching (Bachkirova and Smith, 2015).

Coaching scholars argue that a coach's way of “*being with clients*” (Gendlin, 1969; Linder-Pelz and Hall, 2007; Silsbee, 2008; Divine, 2009; Sieler, 2010; Madison, 2012; Strozzi-Heckler, 2014) rather than their out-of-the-toolbox way of “*doing coaching*” session by session is likely to make a significant difference in how clients feel capacitated to attain goals in coaching. We assume that nonverbal synchrony is one such facet of “being with clients,” which may support their capacity to engage in higher levels of engagement in goal attainment (Grant, 2003; Spence, 2007; Prywes, 2012).

*Hypothesis 2a:*

Higher self-regulation, as operationalized through mood (PANAS, positive and negative affects) and as a result of nonverbal synchrony, predicts a higher client's engagement in goal attainment in coaching.

*Hypothesis 2b:*

Higher self-regulation, as operationalized through a result-oriented problem and self-reflection (RoPS) and as a result of nonverbal synchrony, predicts a higher client's engagement in goal attainment in coaching.

### Working Alliance, Self-Regulation, and Nonverbal Synchrony

In the domain of psychotherapy process research, nonverbal synchrony was found to embody aspects of the therapeutic alliance (Ramseyer and Tschacher, 2011; Altmann et al., 2020; Cohen et al., 2020) and also predicted therapy outcome assessed by pre-to-post symptomatology (Ramseyer and Tschacher, 2011). The In-Sync model (Koole and Tschacher, 2016) has been suggested as a possible theoretical framework for these findings, but other studies failed to confirm a positive association between synchrony and alliance (Paulick et al., 2018b; Schoenherr et al., 2019a,b; Lutz et al., 2020). One of these contradicting studies suggests that the association between nonverbal synchrony and alliance may depend on whether it was assessed from a nomothetic or an idiographic perspective (Ramseyer, 2020a).

**Figure 1:**
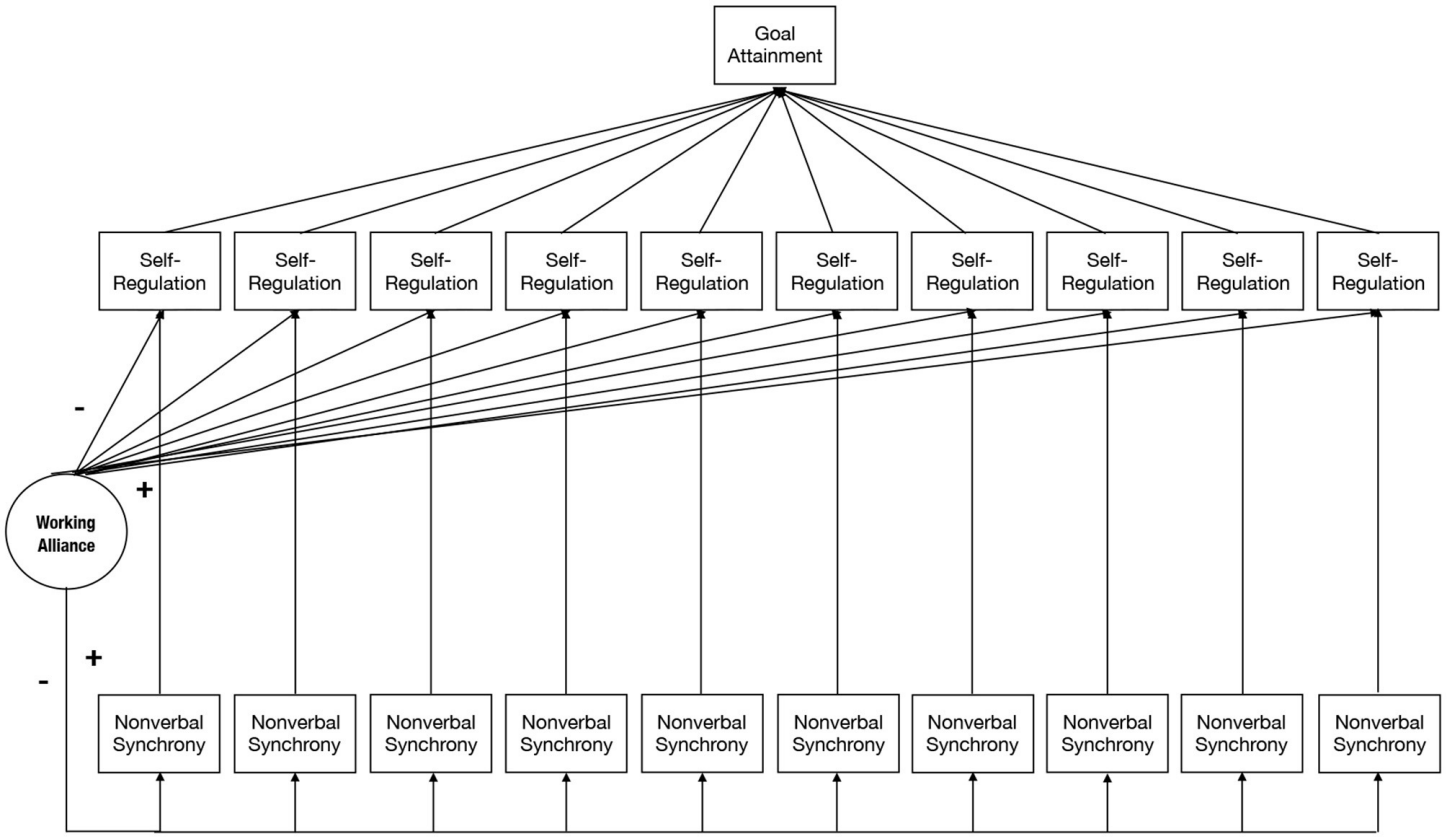
Prediction Model. Prediction model, in which Nonverbal Synchrony represents nonverbal body responses in dyads as measured with MEA; Self-Regulation as measured through positive/negative affect; Working Alliance as measured through Working Alliance Inventory is predicted to moderate the relationship between Nonverbal Synchrony and Self-Regulation as a process. Goal attainment is measured through goal-directed behavior scales and is mediated via Self-Regulation as a process.

In line with the theoretical position that the working alliance is associated with but does not cause coaching outcomes (Graßmann et al., 2020), we propose that working alliance embodies an interpersonal variable with moderator effect. As such, it will strengthen or weaken how clients self-regulate and will be strengthened or weakened by how both a coach and a client dance in the moment to the beats of nonverbal synchrony over time (Figure 1).

*Hypothesis 3:*

3a) Affect balance moderates the direct effects of nonverbal synchrony on a client's self-regulation (operationalized through result-oriented problem and self-reflection, RoPS). 3b) Working alliance moderates the direct effects of nonverbal synchrony on a client's self-regulation (RoPS). 3c) Both affect balance as well as working alliance moderate the direct effect of nonverbal synchrony on a client's self-regulation (RoPS).

## Methods

### Design

This study involved the commitment from several international professional coaching bodies and various organizations working with internal or external coach pools to ensure the enrollment of trained coaches with adherence to at least one professional coaching organization. It was conceptualized to be maximally naturalistic in terms of sample characteristics (professional coaches, common clients, coaching setting) and, also, in terms of cultural diversity (Table 1) to ensure a certain level of generalizability. Coaches were required to deliver workplace coaching with a goal being to identify areas for day-to-day performance improvement. Coaches were thus encouraging clients to develop skill sets to take a proactive role in their workplace development.

**Table 1:** Frequency distribution of sample by country.

	**Frequency distribution**		
**Country**	**Frequency**	**Valid percent**	**Cumulative percent**
Australia	7	3.8	3.8
Austria	2	1.1	4.9
Belgium	4	2.2	7.4
Brazil	4	2.3	9.2
Canada	3	1.6	10.9
Chile	2	1.1	12.0
China	2	1.1	13.0
Czech Republic	4	2.2	15.2
Denmark	2	1.1	16.3
Ecuador	4	2.2	18.5
Egypt	2	1.1	19.6
Finland	2	1.1	20.7
France	1	0.5	21.2
Greece	9	4.9	26.1
Hungary	2	1.1	27.2
India	5	2.7	29.9
Indonesia	4	2.2	32.1
Ireland	2	1.1	33.2
Italy	4	2.2	35.3
Japan	2	1.1	36.4
Kazakhstan	2	1.1	37.5
Lithuania	2	1.1	39.2
Netherlands	22	12	50.5
Poland	3	1.6	52.2
Romania	2	1.1	53.3
Saudi Arabia	21	11.4	64.7
Singapore	1	0.5	65.2
Slovenia	4	2.2	67.4
South Africa	3	1.6	69
South Korea	2	1.1	70.1
United Kingdom	35	19	89.1
USA	20	10.9	100
Total	184	100	

*Frequency indicates the number of participants per country. The Valid Percent column shows the percentage that does not include missing cases. Cumulative Percent adds the percentages of each region from the top of the table to the bottom, culminating in 100*.

**Figure 2:**
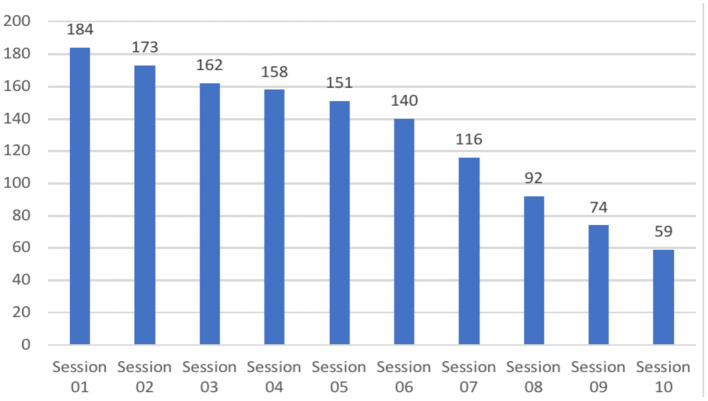
Frequency distribution of session in the data collection phase. Data collection phase lasted from October 2018 through to November 2019. One hundred and eighty four dyads completed 1 session; 59 dyads completed 10 sessions.

**Figure 3:**
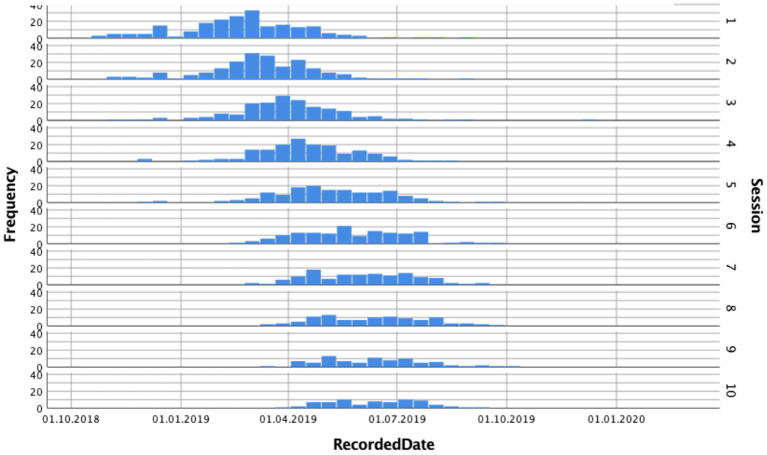
Periodic distribution of coaching sessions. Histogram depicts the perodic distribution of coaching sessions in the period between October 2018 and November 2019.

**Figure 4:**
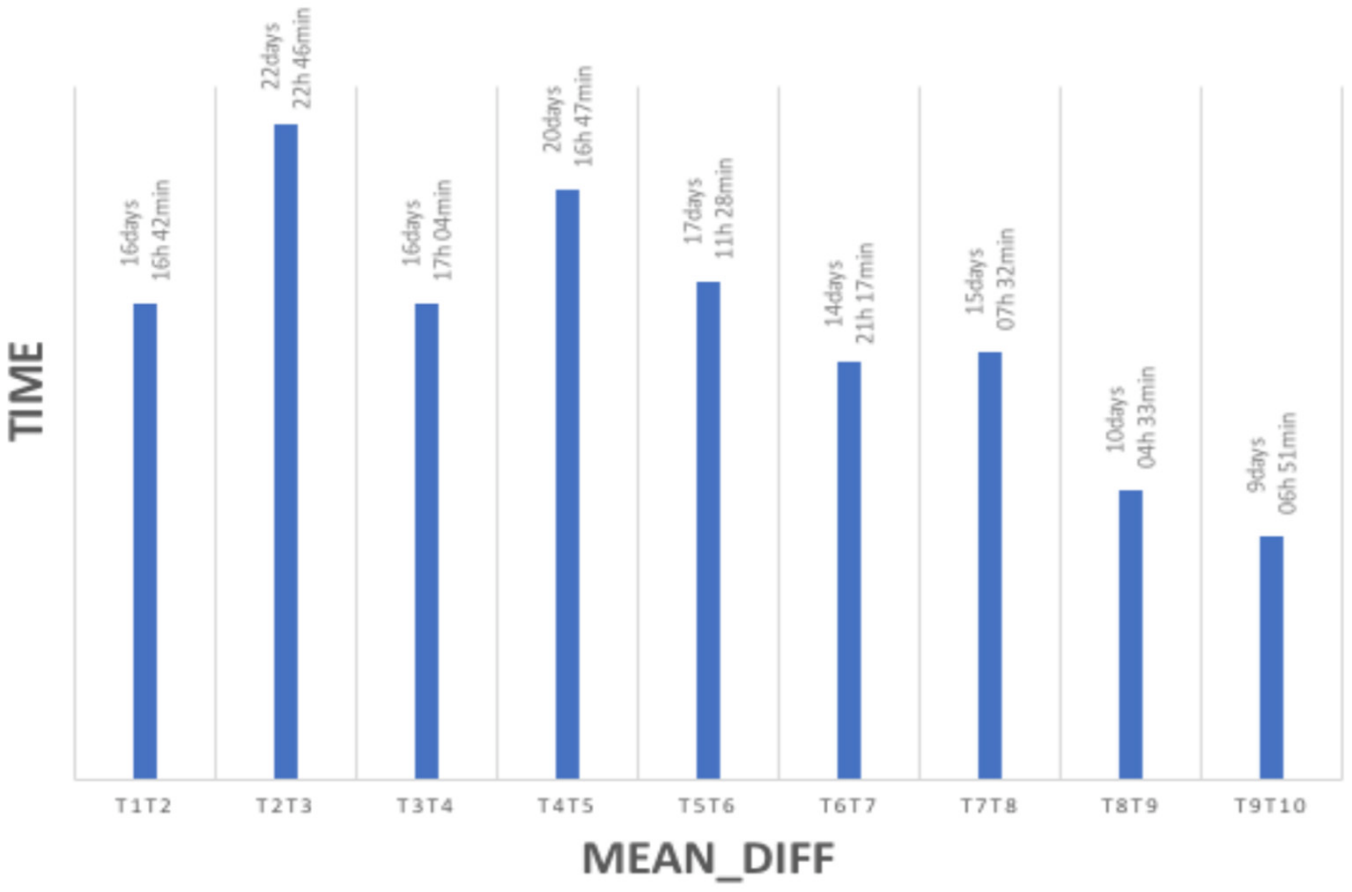
Frequency distribution of session completion by dyads. MEAN_DIFF depicts the average timespan between sessions T1T2, T2T3… TIME indicates the amount of days, hours and minutes of the timespan between sessions.

Conceptually, the study investigated both process measures (i.e., nonverbal synchrony, working alliance, and emotional and cognitive self-regulation) and outcome measures (i.e., goal attainment). It was designed to account for the rich realities of coaching engagements (e.g., participants' choice of frequency of sessions, maximum duration of sessions, themes/goals addressed in coaching, language used in coaching, type of coaching conducted). It comprised up to 10 dyadic coaching interventions, each with a minimum duration of 60 min as is standard in coaching. Yet coach–client dyads were free to choose the number of sessions to be held at their discretion, as is standard in each coach's contracting practice. The observational study design implies that some coaches completed their 10 h of coaching engagement earlier than others. Data for *N* = 184 coach–client pairs from *N* = 99 coaches were collected between October 2018 and November 2019 (Figure 2). Figure 3 shows the distribution of the number of coaching sessions over the data collection phase. Figure 4 depicts the time span between sessions per dyad.

Each coaching session was video recorded by the coaches in the naturalistic setting of the coaching engagement to capture real-time face-to-face interaction processes through body movement for further analysis of nonverbal synchrony data. Where coaches conducted 60-min sessions, they delivered 10 video-taped files while others provided fewer video-recorded sessions where their engagement was to end sooner according to the coaching contract. For the purposes of automated video-analysis in coaching as a non-clinical helping intervention, it was assumed that the clients, who were not subject to inclusion or exclusion criteria based on diagnosis (e.g., psychosis, substance dependency), would have a normal capacity to synchronize nonverbally as observed in student dyads (Lozza et al., 2018; Fujiwara et al., 2019; Dunbar et al., 2020).

Video-data collection and video-file transmission were conducted in compliance with GDPR rules and regulations as defined in the ethics approval awarded by the research institute. Data on a client's self-report process measures were collected through online questionnaires within 24 h after each session. Questionnaires were made available in validated English language versions. Goal attainment questionnaires for client's self-reports were administered once 3 months after the coaching engagement was completed. This design required that all the participants sign a written informed-consent form prior to enrollment in this study. Links to clients' self-report questionnaires were transmitted to the clients via their coach to ensure the clients' privacy and data safety. The coaches were not required to complete any self-reports. Comprehensively, the data presented here may thus be considered to be a convenience sample (Jager et al., 2017).

### Recruitment

The minimum requirement for coach–client dyad recruitment (*N* = 150) targeted to establish statistical relevance led to the enrollment of 184 coach–client pairs (*N* = 184). The recruitment phase ran from June 2018 through January 2019. It involved a rigorous selection process of individual in-depth interviews with the coaches, each lasting 60 min to contract their enrollment. The interviews were conducted by the corresponding author of this article. The coaches were guided to a dedicated research website to access detailed technical instructions for the video recording and a file-transfer process. Additionally, the procedures as well as the specific IT support framework provided transparently for participation (www.coachingpresenceresearch.com). To enroll the 184 coach–client pairs, the project was presented at several professional conferences around the globe between 2017 and 2018. The coaches were asked to recruit their clients for the purposes of this study, and client data were coded in all steps throughout the research to ensure client anonymity.

### Participants

**Figure 5:**
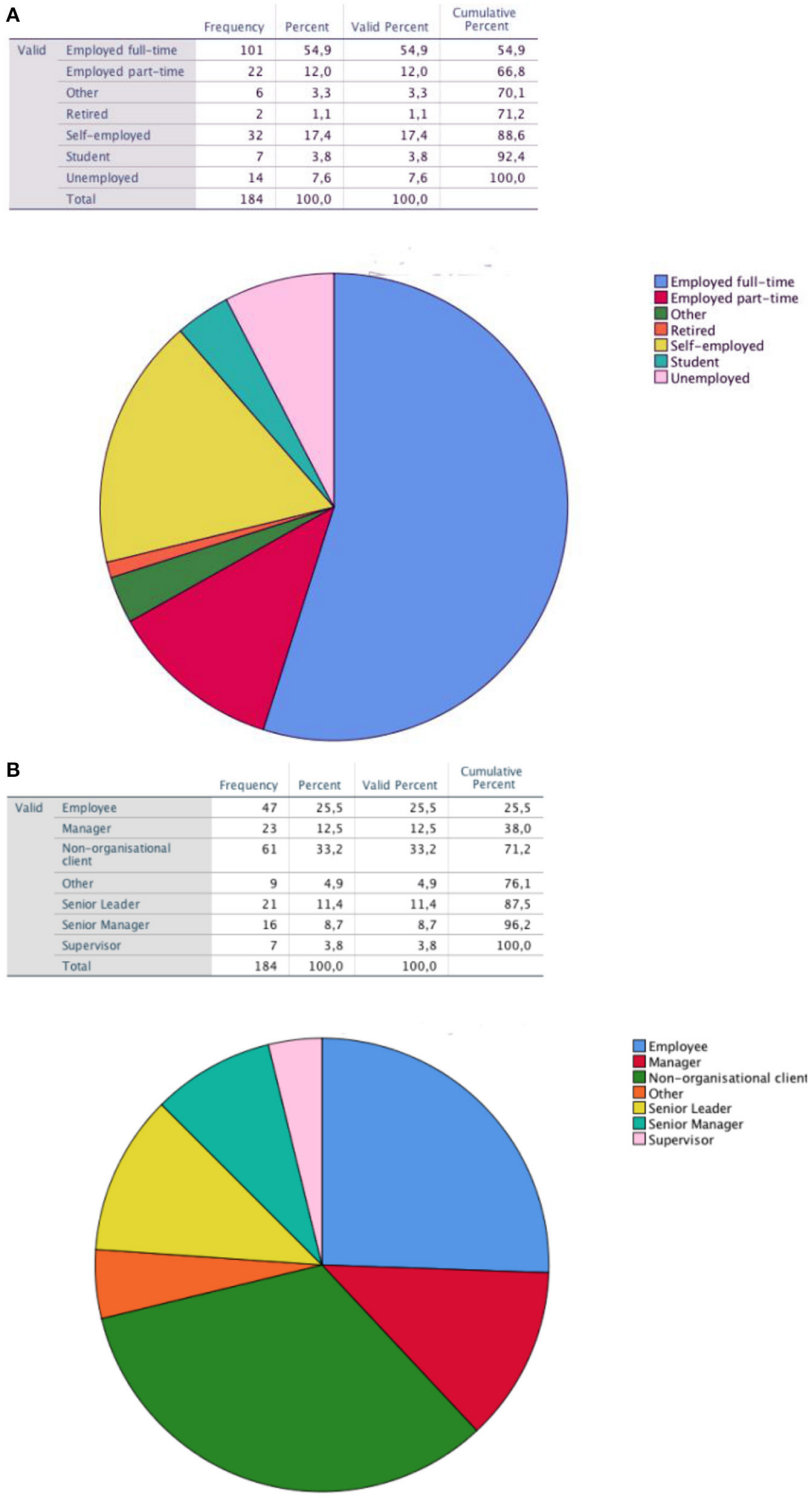
**(A)** Client employment category. The pie chart depicts the distribution of clients' category of employment by 7 categories ranging from full-time employment to unemployment. **(B)** Client position in organization. The pie chart depicts the distribution of clients' level of employment by 7 categories ranging from employee to supervisor.

The coaches (*N* = 99) were predominantly female (*n* = 79; 79.8% vs. male *n* = 20; 20.2%) while the clients (*N* = 184) had a more balanced distribution in terms of gender (male *n* = 66; 35.9% vs. female *n* = 118; 64.1%). The number of the clients per coach varied: *N* = 31 coaches worked with *n* = 1 client, *N* = 55 coaches worked with *n* = 2 clients, *N* = 8 coaches worked with *n* = 3 clients, and *N* = 5 coaches worked with *n* = 4 clients. Further characteristics in terms of age were more or less mixed, as could be expected based on the recruitment strategy selected for this research project. Figures 5A,B show the distribution of the clients' employment categories and levels of employment in organizations.

**Figure 6:**
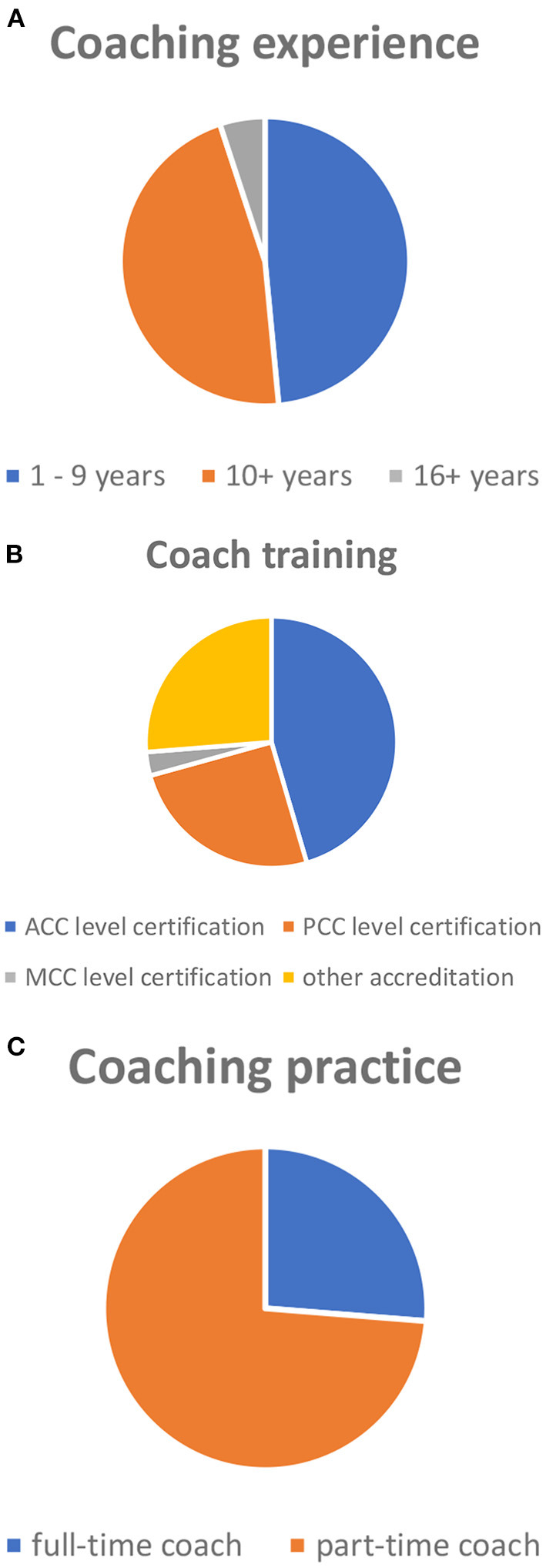
**(A)** Coach participation based on level of experience. Coaching experience prior to enrollment was defined by three categories: 1–9 years, 10+, and 16+ years. **(B)** Coach participation based on level of training. Coaching training requirements as based on ICF's (International Coaching Federation) certification levels: ACC level certification requires 60+ h of coach-specific training and 100+ h of coaching experience; PCC level certification requires 125+ h of coach-specific training and 500+ h of coaching experience; MCC level certification requires 200+ h of coach-specific training and 2,500+ h of coaching experience. “other accreditation” refers to any other coach-specific training outside ICF's scope of accreditation. **(C)** Coach participation based on level of coaching practice. Coaching training requirements as based on coaches' full-time or part-time professional practice.

The study was designed to represent qualified professional coaches (Figures 6A–C) specialized in leadership coaching, career management, and business coaching. The coaches were selected on the basis of specific criteria (i.e., years of coaching experience, levels of coaching training, and levels of professional practice) that were defined as sufficient for the targeted quality of this study.

### Instruments

#### Motion Energy Analysis (MEA)

MEA (Ramseyer and Tschacher, 2011; Ramseyer, 2020b) is based on video recordings where “motion energy” is defined as the difference in gray scale pixels between consecutive video frames (Grammer et al., 1999), the working principle of computer-vision motion detection (Sonka et al., 2007).

#### Quantification of Nonverbal Synchrony

**Figure 7:**
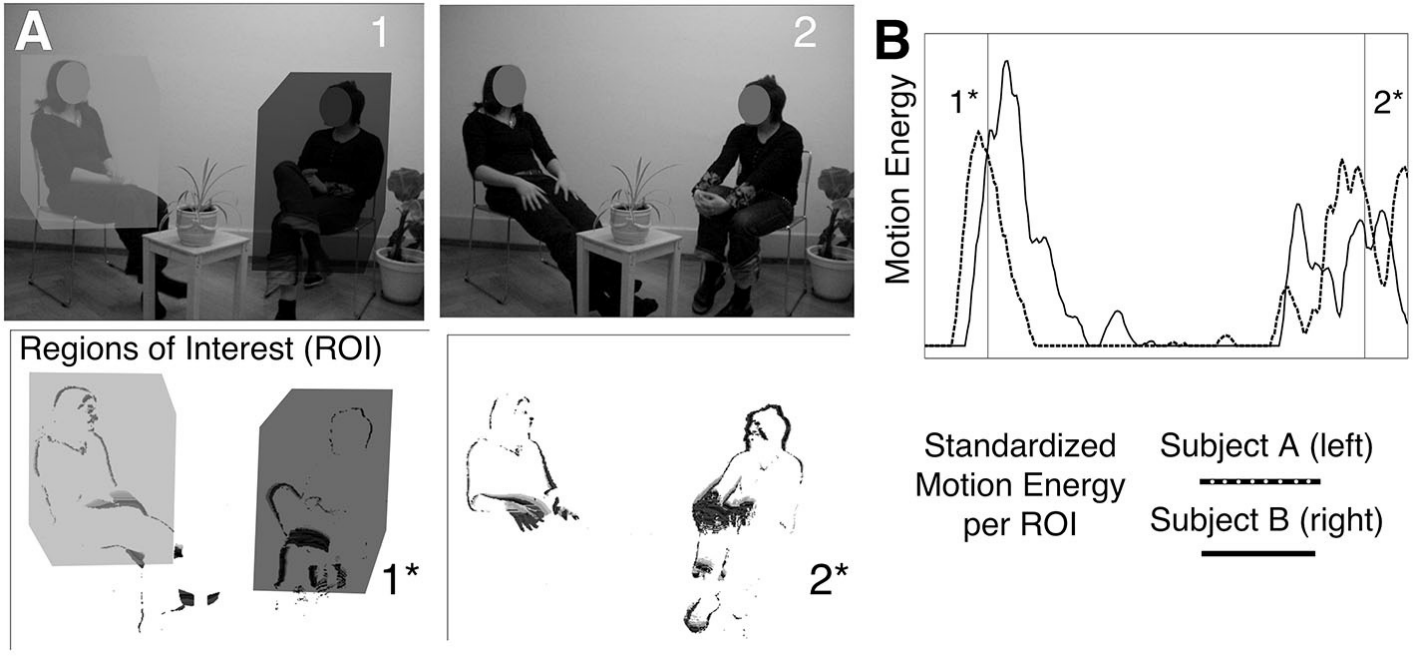
Motion Energy Analysis (MEA): Panel **(A)**: Top row = Original Movie (1, 2). Bottom Row = Frame-Differenced Pictures (1*, 2*). Panel **(B)**: Motion Energy per Region of Interest (ROI).

Provided that the camera position remains fixed, and that the background as well as lighting conditions are kept constant, frame-by-frame changes are indicative of body movement occurring in predefined regions of interest (ROI) (Ramseyer, 2020b). For the purposes of the present study, three body parts were defined as ROIs for each interactant: head to shoulder, upper body from shoulder to hips, lower body to feet. In the remainder of this article, we focus on an overall region comprised of the sum of all three regions per person. This simplification is based on previous work, employing full-body ROIs (Ramseyer and Tschacher, 2011) and, more specifically, because we decided to keep to potential number of analyses lower than would be the case with three ROIs. It should be noted that the frame-differencing algorithm captures the dynamics and extent of movement rather than the qualitative aspect or the localization of specific movements and gestures (see Figure 7).

In the present study, MEA (Version 4.03; see www.psync.ch) was used to analyze the first 25 min of a session. We arrived at this limit of 25 min because we wanted to ensure comparability across varying session duration and because previous work has reported very high associations between 15-min segments and whole sessions (Ramseyer and Tschacher, 2011; Paulick et al., 2018a; Altmann et al., 2020). After trimming of initial video sequences (e.g., first 3 min of settling in were removed) a frame rate and video-codec conversion to codec h.264 at 25 frames/s were performed with open source software FFmpeg for video files that were not based on codec h.264. The aim was to ensure maximum comparability across different recording settings. Furthermore, prior to running the rMEA analysis, a full-time quality check spanning 3 months (i.e., June to August 2019) was conducted by the first author, following the four-eye principle with the IT specialist hired for managing and archiving video data. This analysis involved the thorough visual inspection of each video for potential anomalies (e.g., change in lighting, resolution quality, other variations in the technical quality of videos) because the coaches had been free to use various types of video recording devices (i.e., iPhone, video camera, PC camera). This screening ensured consistent video quality for the naturalistic setting of this study. Out of a total of *N* = 1,323 video files, *n* = 13 files were excluded because of low quality that could have led to erroneous analytical results.

The *R*-package rMEA (Kleinbub and Ramseyer, 2020) was used for the statistical calculation of an index of synchrony expressed as the coordination of movements between a coach and a client. Simultaneous as well as lagged cross-correlations of the time-series of each dyad were calculated (function *MEAccf* in rMEA) for lags up to ±5 s (*lagsec* = 5). The resulting correlation coefficients capture the simultaneous as well as the time-lagged synchronization of both a dyad member's body movements. The absolute values of these correlation coefficients are transformed to Fisher's *Z* values, which are averaged in order to obtain a grand-mean value of synchrony for each session (*r2Z* = T; *ABS* = T). To account for the potential non-stationarity of the data, each 25-min conversation was split up into non-overlapping segments of 60-s duration, and the cross-correlations were computed separately in each segment (*winSec* = 60, *incSec* = 60). The overall index of synchrony in one session of coaching is thus given by the absolute grand mean of cross-correlations over all segments in this conversation (Ramseyer, 2020b). We chose these parameters based on the first empirical study using MEA (Ramseyer and Tschacher, 2011) because, in this way, we are better able to compare the psychotherapy samples and the coaching samples from the present study. The parameters reported above have been successfully used in a number of studies (Ramseyer and Tschacher, 2011; Dean et al., 2018; Galbusera et al., 2018; Asher et al., 2020; Cohen et al., 2020; Ramseyer, 2020a), and the extended graphical inspection possibilities available in rMEA suggested good suitability of said parameters for our coaching sessions (rMEA functions: *MEAheatmap, MEAlagplot, MEAdistplot*).

#### Key Video Specifications

The present study was conducted in line with the specifications defined for MEA (Ramseyer, 2020b) and successfully applied in numerous studies (see www.psync.ch for details). For the coaching sessions, the first 3 min of each interaction were skipped to remove sections that concerned the preparatory process of settling in (e.g., showing clients to the room; starting the recording equipment; moving chairs about, etc.).

#### Pseudosynchrony

In a further methodological step, we applied surrogate analysis by randomly shuffling the time series across the participants as suggested by Kleinbub and Ramseyer (2020) —between shuffling; *shuffle* = 5,000—generating “pseudo-interactions” (Bernieri et al., 1988). Pseudosynchrony was then computed from each pseudo-interaction as described above. Comparing “genuine” synchrony with the distribution of pseudosynchrony yields an effect size of nonverbal synchrony (Ramseyer and Tschacher, 2010; Moulder et al., 2018).

#### Working Alliance (WAI)

We employed the revised WAI-SR version (Hatcher and Gillaspy, 2006) of the 12-item (7-point Likert scale) Working Alliance Inventory originally developed by Horvath and Greenberg (1989) to measure goal setting, the bond, and task orientation in the coaching relationship. The WAI-SR has high internal consistency; Cronbach's *a* of the subdomains ranges from 0.81 to 0.90, and Cronbach's *a* of the total score is 0.91. The WAI-SR has high reliability, with test-retest reliability of 0.93 [95% CI 0.83, 0.97]. With regard to construct validity, the WAI-SR correlates well with other therapeutic alliance measures. Furthermore, higher scores on the WAI-SR are associated with better treatment outcomes (Flückiger et al., 2018), confirming the WAI-SR's construct validity in accordance with Bordin's (1979) theory.

#### Result-Oriented Problem and Self-Reflection (RoPS)

The RoPS (Greif and Berg, 2011) scales (27 items, four-point Likert scale) were used to assess various aspects of goal reflection (RoPS-GR; sample item: “The last time I thought about myself and my goals, I considered how much I was willing to invest for these goals.”), reflection of self-organization (RoPS-SO); sample item: “Within the last few weeks, I thought about my personal standards, needs, and goals, and developed a plan on how to reach them,” and reflection of concrete changes from session to session (RoPS-CC); sample item: “The last time I thought about a special problem, I resolved to concretely change my behavior so that I might be better able to handle the problem in the future.” Mean reliability of the RoPS scales range between *a* = 0.70 and *a* = 0.80, and may thus be considered acceptable.

#### Affective Experience (PANAS)

In order to assess the current distribution of positive and negative emotions, we used the 20-item positive and negative affect scale (PANAS) (Watson et al., 1988), which reliably measures two primary dimensions of mood: positive and negative affects (Cronbach's *a* ranging from 0.86 to 0.90 for *positive affect* and from 0.84 to 0.97 for *negative affect*). The 10-item scales for each affect schedule (words describing various emotions ranging from happy to scared) are internally consistent and have excellent convergent and discriminant validity with lengthier measures of the underlying mood factors (Watson et al., 1988). A third factor affect balance (PANAS-AB) was calculated, using a variation of the method by Koydemir et al. (2013) by quantifying the difference between positive and negative affects.

#### Goal Attainment

The measures used to assess clients' levels of engagement in goal-directed behavior 3 months after coaching was assesses with six constructs: *Perceived Goal Competence* relating to the clients' feelings able to act effectively to attain important goals consists of four items (e.g., “I feel I am able to meet the challenge of attaining my goal.”) as adopted from Williams and Deci (1996) with a Cronbach's *a* of 0.72; *Planning* relating to the clients' cognitive capacity to specify steps (i.e., how when and where) required to attain goals consists of four items (e.g., “I have identified specific behaviors that will help me achieve my goal”) as developed by Prywes (2012), with a Cronbach's *a* of 0.74; *Conscientiousness* relating to the clients' propensity for planning and being purposeful consists of 10 items (e.g., “I carry out my plan.”) as adopted from Markey and Markey (2009), with a Cronbach's *a* of 0.84; *Goal Commitment* relating to the clients' determination to reach a goal consists of five items (e.g., “I am strongly committed to pursuing this goal.”) as adopted from Klein et al. (2001), with a Cronbach's *a* of 0.72; *Goal Self-Concordance* relating to the clients' enduring interest and motivation to pursue self-set goals consists of four items (e.g., “You strive for this goal because of the enjoyment or stimulation that goal provides you.”) as adopted from Sheldon and Houser-Marko (2001), with a Cronbach's *a* of 0.52; *Goal Stability* relating to the clients' aspirations over the course of the study consists of three items (“My interest in this goal did not change significantly over the past 3 months.”), as developed by Prywes (2012), with a Cronbach's *a* of 0.77. Our principal components analysis of all subscales indicated that, by excluding the “goal stability” items, a single-factor solution explained 60.29% of the variance, with a Cronbach's *a* of 0.83. The remaining five subscales intercorrelated between *r* = 0.423 and 0.637; thus, we limit our report to this averaged overall goal scale, consisting of 27 items.

### Statistical Analysis

As discussed in the section of study questions and recruitment, the analysis reported here was conducted on a sample of naturalistic coach–client processes. The sample size had been determined according to previous work with MEA, providing medium-effect sizes for associations with process measures and psychotherapy outcome (*r* = 0.30; Ramseyer and Tschacher, 2011). Given the open nature of recruitment, a minimum of 150 dyads was targeted as this would have resulted in a value of 1 – β = 0.97 for the main effect, showing an association between synchrony and self-regulation.

Given the exploratory nature of the study, statistical analysis was conducted in a step-up fashion, moving from simple data models to sophisticated approaches. First, comparisons across groups were performed with simple *t-*tests and ANOVAs. Next, the temporal aspects of coaching, synchrony, self-regulation, and outcome were computed by multilevel modeling, using the module GAMLj (Galluci, 2021) for the software package jamovi (The jamovi project, 2020). The data were structured in three levels: sessions (level 1; *n* = 1 to 10) were nested in dyads (level 2; *N* = 184), nested in coaches (level 3; *N* = 99). Dependent variables in multilevel models were either “result-oriented problem and self-reflection” (RoPS) or “affect balance” (PANAS-AB). Fixed effects were “nonverbal synchrony” (MEA), “working alliance” (WAI), and either “result-oriented problem and self-reflection” (RoPS) or “affect balance” (PANAS-AB). Additional outcome factors were the levels of “goal attainment” (GOAL). Random effects were “intercepts” of clients and coaches. Several multilevel models were constructed by subsequently adding predictors in order to explore the effects of “synchrony” and “process measures” on the cognitive and affective aspects of “self-regulation.” The model fit was compared according to the corrected Akaike information criterion (AICc). Degrees of freedom were calculated, using the Satterthwaite method available in GAMLj (Galluci, 2021). Interaction effects were entered for the assessment of Hypothesis 3, predicting moderation effects of WAI on the association between synchrony and self-regulation (RoPS and PANAS). See Figure 1 for the *Prediction Model*.

In a further explorative step, the temporal associations across coaching sessions were modeled, using a network approach (Epskamp, 2020; Jordan et al., 2020), which has recently been applied to various fields of psychology, such as clinical psychology (Lutz et al., 2018; Kaiser and Laireiter, 2019) or patient-physician interactions (Hamel et al., 2020). In this modeling approach, a phenomenon is seen as a network of specific elements (e.g., symptoms, factors) that dynamically interact and impact one another over time. As such, observed variables in this dataset (synchrony, result-oriented problem and self-reflection, mood) are conceptualized as causal agents in a dynamic interplay over time. We used the package *mlVAR* (Epskamp et al., 2019) in the open software *R* (Version 3.4.0; Team, 2008) to estimate networks for the entire sample (all clients), and networks of subgroups defined by the clients' levels of goal attainment after coaching. Three equal groups were defined based on GOAL-scores, each containing ~33% of the clients (high GOAL, mid GOAL, low GOAL). The clients without GOAL assessments (*n* = 8) were not included in the network analyses of the subgroups.

## Results

### General Characteristics and Outcomes of Coaching

The complete sample of 184 dyads attended between 1 and 10 sessions of coaching (*M* = 7.13; *SD* = 2.88; median = 8). Of the 176 dyads reporting their level of goal attainment (GOAL) 3 months after the completion of the coaching engagement, the majority specified a high level of success from their coaching sessions, as indicated by their positive perspectives on goals (*M* = 5.66; *SD* = 0.72). GOAL assessment was not related to the number of sessions attended [*r* (175) = 0.015; *p* = 0.841]. Self-reported mood (PANAS) was predominantly positive (*M*_pos_ = 37.44; *SD*_pos_ = 8.00; *N* = 1,312; *M*_neg_ = 16.61; *SD*_neg_ = 6.54; *N* = 1,312), and there was a significant increase in the positive mood across coaching [*session* = 0.44; *t* (1,175.2) = 8.12; *p* < 0.001; ICC = 0.619], while the negative mood showed a significant temporal decrease [*session* = −0.38; *t* (1,173.4) = −7.89; *p* < 0.001; ICC = 0.579]. Affect balance (PANAS-AB) was very similar to the positive mood scale: There was an increase over time [*session* = 0.817; *t* (1,177) = 9.82; *p* < 0.001; ICC = 0.594].

Solution-oriented problem and self-reflection (RoPS) were high (*M*_*tot*_ = 3.13; *SD*_tot_ = 0.58; *N* = 1,312) and significantly increased with coaching [*session* = 0.07; *t* (1,177.6) = 16.88; *p* < 0.001; ICC = 0.588]. Subscales relating to the reflection of self-organization (*M*_SO_ = 3.08; *SD*_SO_ = 0.65), reflection of concrete changes (*M*_CC_ = 3.17; *SD*_CC_ = 0.61), and goal-reflection (*M*_GR_ = 3.20; *SD*_GR_ = 0.66) all showed similarly high levels, and all subscales had a significant effect of time (*session* = 0.065–0.071, all *p'*s <0.001). A comparable result was found for the overall working alliance (WAI), which was reported to be high (*M*_tot_ = 4.40; *SD*_tot_ = 0.59; *N* = 1,312) and also significantly increased across sessions over time [*session* = 0.04; *t* (1,159.5) = 11.74; *p* < 0.001; ICC = 0.702]. The subscales of bond (WAI-B), task orientation (WAI-T), and the goal setting (WAI-G) all indicated similar effects already present in the overall scale: The effect of task orientation was highest (*M*_T_ = 4.40; *SD*_T_ = 0.59; *N* = 1,312), followed by the effect of bond (*M*_B_ = 4.40; *SD*_B_ = 0.59; *N* = 1,312) and the effect of goal setting (*M*_G_ = 4.40; *SD*_G_ = 0.59; *N* = 1,312). All subscales increased across sessions over time (*session* = 0.032–0.052; all *p's* < 0.001).

### Nonverbal Synchrony

Nonverbal synchrony was clearly different from pseudosynchrony (coincidence): The comparison with 5,000 pseudodyads was highly significant [*t* (382.8) = 9.10; *p* < 0.001], and this difference had a medium effect size (Cohen's *d* = 0.67). Across all subjects, synchrony decreased over time [*session* = −0.001; *t* (1,161.2) = −4.09; *p* < 0.001; ICC = 0.625].

Process measures (WAI, RoPS, PANAS) were highly correlated (*r* = 0.513–0.593), while session-level synchrony was almost independent of each of the specific process measures (*r* = −0.062–0.013). See Table 2 for details.

**Table 2:** Session-report scores from post-session questionnaires.

	**Sample (*****N*** **= 184) 64.1% female**											
			**RoPS**			**WAI**			**PANAS**			
	**M**	**SD**	**GR**	**SO**	**CC**	**T**	**B**	**G**	**Pos**	**Neg**	**AB**	**Sync**
RoPS-GR (184)	3.135	0.484										
RoPS-SO (184)	3.069	0.513	0.845									
RoPS-CC (184)	3.152	0.463	0.832	0.855								
WAI-T (184)	4.200	0.627	0.633	0.693	0.760							
WAI-B (184)	4.453	0.540	0.448	0.495	0.542	0.689						
WAI-G (184)	4.347	0.620	0.585	0.627	0.663	0.890	0.704					
PAN-P (184)	37.208	6.584	0.603	0.623	0.674	0.642	0.439	0.538				
PAN-N (184)	16.800	5.630	−0.181	−0.215	−0.242	−0.226	−0.179	−0.165	−0.289			
PAN-AB (184)	20.829	11.947	0.439	0.463	0.516	0.495	0.383	0.394	0.858	−0.777		
Synchrony (173)	0.121	0.022	−0.050	−0.009	−0.085	−0.040	−0.028	−0.013	0.007	−0.111	0.043	
GOAL (176)	5.659	0.716	0.457	0.445	0.416	0.413	0.326	0.377	0.395	−0.326	0.448	0.015

*Session-report scores from post-session questionnaires (WAI), result-oriented problem and self-reflection (RoPS), positive and negative affect, affect balance (PANAS), and nonverbal synchrony in all available clients (overall). Outcome GOAL after completion of sessions. Correlations of process-measures based on average scores per client, correlations with GOAL based on averages across all sessions*.

At the level of simple associations, goal attainment was not found to be related to the development of synchrony across sessions, given that the slope of synchrony, average synchrony, and initial synchrony were uncorrelated with goals (all *r* < 0.10; *p* = n.s.). On the other hand, all three self-report process measures (RoPS, WAI, PANAS) predicted the attainment of goals (*r* = 0.326–0.457), as reported in Table 2.

### Hypotheses

*Hypothesis 1a*—which predicted a positive relationship between nonverbal synchrony and emotional self-regulation—could not be confirmed: The mixed model showed no direct influence of synchrony on affect balance, PANAS-AB [*t* (1,250.8) = 0.007; *p* = 0.994] The assessment of Hypothesis 1b resulted in a similar picture, namely that synchrony alone did not predict overall cognitive self-regulation (total RoPS), [*t* (1,265.9) = 1.498; *p* = 0.134], see **Table 4** for further details on mixed models.

Regarding *Hypotheses 2a* and *2b*, we found that all self-report process measures were associated with goal attainment: As reported in Table 2, the scales of RoPS showed positive associations with goal attainment (*r* = 0.416–0.457), which was also the case for working alliance (*r* = 0.326–0.413). Positive affect as well as affect balance predicted higher goal attainment (*r* = 0.395; 0.448), while negative affect showed a negative association with goal attainment (*r* = −0.326).

**Figure 8:**
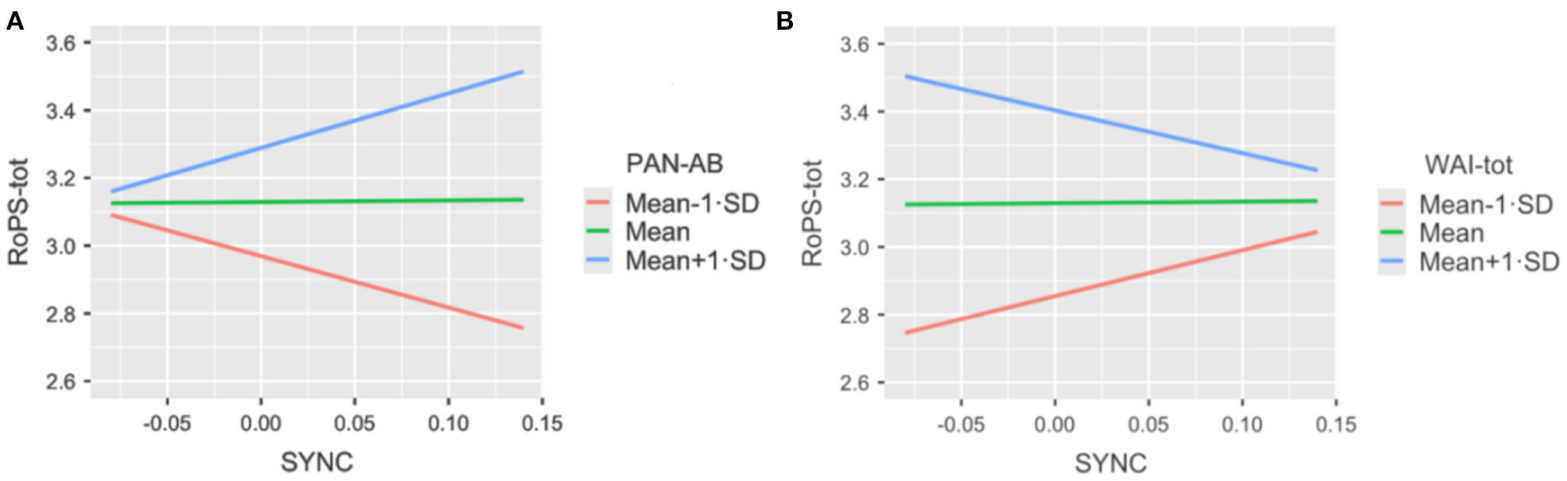
**(A,B)** Sync PAN AB. **(A)** Interaction between synchrony (SYNC, X-axis), affect regulation (PAN-AB; color of slopes) and cognitive self-regulation (RoPS-tot, Y-Axis). **(B)** Interaction between synchrony (SYNC, X-axis), working alliance (WAI-tot; color of slopes) and cognitive self-reflection (RoPS-tot, Y-Axis).

These effects partially fall in line with *Hypothesis 3*: While working alliance was not directly associated with nonverbal synchrony, we found interaction effects (moderation) of WAI and emotional self-regulation (PANAS) on the association of nonverbal synchrony with cognitive self-regulation (RoPS): In dyads reporting high levels of affect balance (PAN-AB), nonverbal synchrony was positively associated with cognitive self-regulation (RoPS-tot), while the reverse was true for dyads reporting low levels of affect balance [*t*(1,275) = 2.895; *p* = 0.004] (Figure 8A). In contrast, in dyads reporting high levels of working alliance (WAI-tot), synchrony was negatively related to cognitive self-regulation (RoPS-tot), while the reverse was true for dyads reporting low levels of working alliance [*t*(1,269.6) = 2.491; *p* = 0.013] (Figure 8B).

We further assessed these interaction effects for moderation (Omnibus Tests) and found that for WAI, levels 1 SD below average (mean – 1*SD*) contributed most to the interaction (*Est* = 1.572; *SE* = 0.718; *t* = 2.190; *p* = 0.029), while, for PANAS-AB, levels 1 SD above average (mean + 1 SD) contributed most to the interaction (*Est* = 1.8; *SE* = 0.721; *t* = 2.587; *p* = 0.010). In other words: WAI moderated the effect of synchrony on cognitive self-regulation (RoPS): In clients with low WAI, more synchrony was associated with higher RoPS, while, in clients with high WAI, less synchrony was associated with higher RoPS. The reverse was true for affect balance: In clients with high affect balance, higher synchrony predicted higher RoPS, while, in clients with low PANAS-AB, low synchrony predicted higher RoPS. These interaction effects were not found in alternative models with either PANAS or WAI as dependent variables (see **Table 4** for further details).

### Temporal Relationships and Network Model Analyses

**Figure 9:**
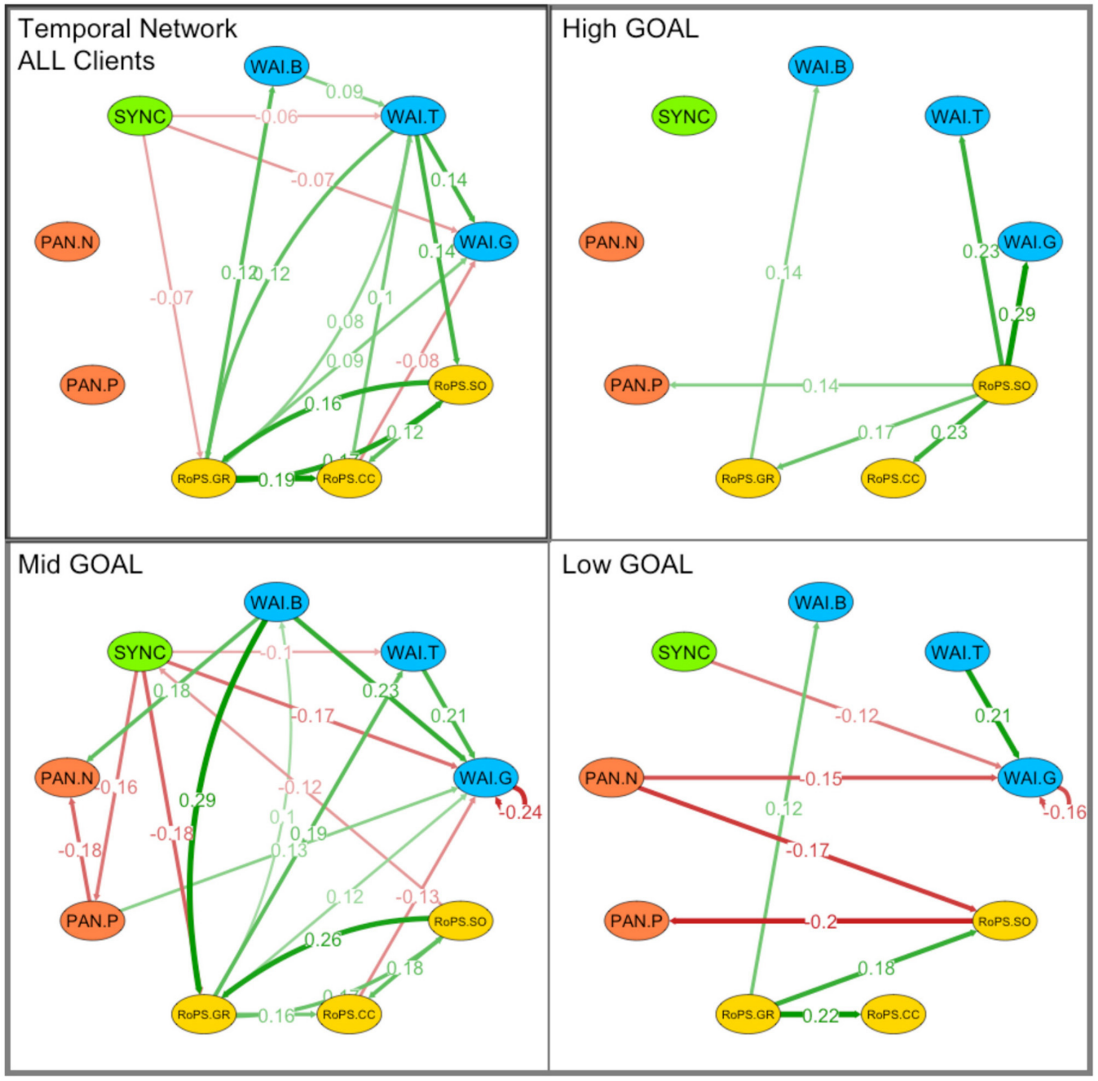
Network analyses: Temporal associations between synchrony (SYNC), working alliance (WAI), affective experience (PAN), and cognitive self-regulation (RoPS). Associations highlight nodes between the following variables: WAI.B, Bond; WAI.T, Task Setting; WAI.G, Goal Orientation; RoPS.SO, Self-Organization; RoPS.CC, Concrete Changes; RoPS:SO, Self-Organization; PAN.P, Positive Affect; PAN.N, Negative Affect; Associations are clustered by “ALL Clients” as well as groups of High GOAL attainment, Mid GOAL attainment, and Low GOAL attainment. Green-colored connections: Positive associations. Red-colored connections: Negative associations. Thickness of lines: Strength of association. Only significant associations depicted.

In a further step, we explored the temporal relationships between nonverbal synchrony and process measures by applying network model analysis (Bringmann et al., 2013; Epskamp, 2020). We first applied a network model to the complete sample, using all available process measures from the post-session self-reports (all scales of WAI, RoPS, PANAS) and the synchrony scores extracted from the videos with MEA (overall SYNC). For the temporal model (Figure 9 Panel “ALL Clients”), the strong positive associations (green arrows) within self-report measures are easily visible (connections between the same-colored circles), as well as further notable temporal associations between scales (lines between different-colored circles): Nonverbal synchrony has negative associations (red arrows) with RoPS and WAI, i.e., lower synchrony in the previous session predicted higher goal self-reflection (RoPS-GR), as well as higher goal orientation (WAI-G), and task setting (WAI-T). The only other negative association was from concrete changes (RoPS-CC) to goal orientation (WAI-G), where less concrete changes predicted higher goal orientation. The most relevant nodes in terms of in and out strength (the number of significant associations with other nodes) are WAI-T and RoPS-GR: They receive (*in strength* = 2) and send (*out strength* = 4 WAI-T; 3 RoPS-GR) positive associations (green arrows) across time. Another relevant node was WAI-G, receiving four associations (two positive and two negative ones). Synchrony has a total of three negative outgoing connections, two with WAI (WAI-T; WAI-G) and one with RoPS (RoPS-GR). In terms of connection strength (thickness of lines), the strongest predictors are found within RoPS itself and between RoPS and WAI. One example is the positive association between goal self-reflection (RoPS-GR) and bond (WAI-B; *r* = 0.122; *p* < 0.001). All nodes and associations related to the models with synchrony are provided in Table 3.

**Table 3:** Parameter Estimates (and Standard Errors) for Network Models.

**Variable**	**Analysis**	**Parameters**				
**Global model**		**From**	**To**	**Fixed**	***SE***	***p***
	Temporal	SYNC	WAI-T	−0.061	0.027	0.023
		SYNC	WAI-G	−0.075	0.033	0.023
		SYNC	RoPS-GR	−0.069	0.035	0.048
		1	2	*pcor*	*p* 1 -> 2	*p* 1 < - 2
	Contemporaneous	-	-	-	-	-
	Between-network	SYNC	RoPS-CC	−0.148	0.108	0.046
**Outcome Level**		**Grouped by GOAL Outcome**				
		**From**	**To**	**Fixed**	***SE***	***p***
High GOAL	Temporal	-	-	-	-	-
		1	2	*pcor*	*p* 1 -> 2	*p* 1 < - 2
	Contemporaneous	-	-	-	-	-
	Between-nework	SYNC	WAI-B	−0.257	0.015	0.027
**Outcome Level**		**From**	**To**	**Fixed**	***SE***	***p***
Mid GOAL	Temporal	SYNC	WAI-G	−0.165	0.073	0.023
		SYNC	RoPS-GR	−0.178	0.067	0.008
		SYNC	PAN-P	−0.158	0.065	0.015
		RoPS-SO	SYNC	−0.118	0.060	0.048
		1	2	*pcor*	*p* 1 -> 2	*p* 1 < - 2
	Contemporaneous	SYNC	WAI-B	−0.092	0.123	0.050
		SYNC	WAI-T	0.105	0.055	0.033
		SYNC	RoPS-SO	0.105	0.057	0.026
	Between-network	SYNC	PAN-N	−0.353	0.001	<0.001
**Outcome Level**		**From**	**To**	**Fixed**	***SE***	***p***
Low GOAL	Temporal	SYNC	WAI-G	−0.117	0.053	0.029
		1	2	*pcor*	*p* 1 -> 2	*p* 1 < - 2
	Contemporaneous	-	-	-	-	-
	Between-network	SYNC	RoPS-CC	−0.356	0.006	0.008

*Parameter Estimates for the associations between synchrony and WAI, PANAS, RoPS (N = 176). Only significant associations involving synchrony are shown. WAI, Working Alliance Inventory; PANAS, Positive And Negative Affect Scales; RoPS, Result-Oriented Problem- And Self-Reflection Scales; SYNC, Nonverbal Synchrony; SE, Standard Error; pcor, partial correlation*.

**Figure 10:**
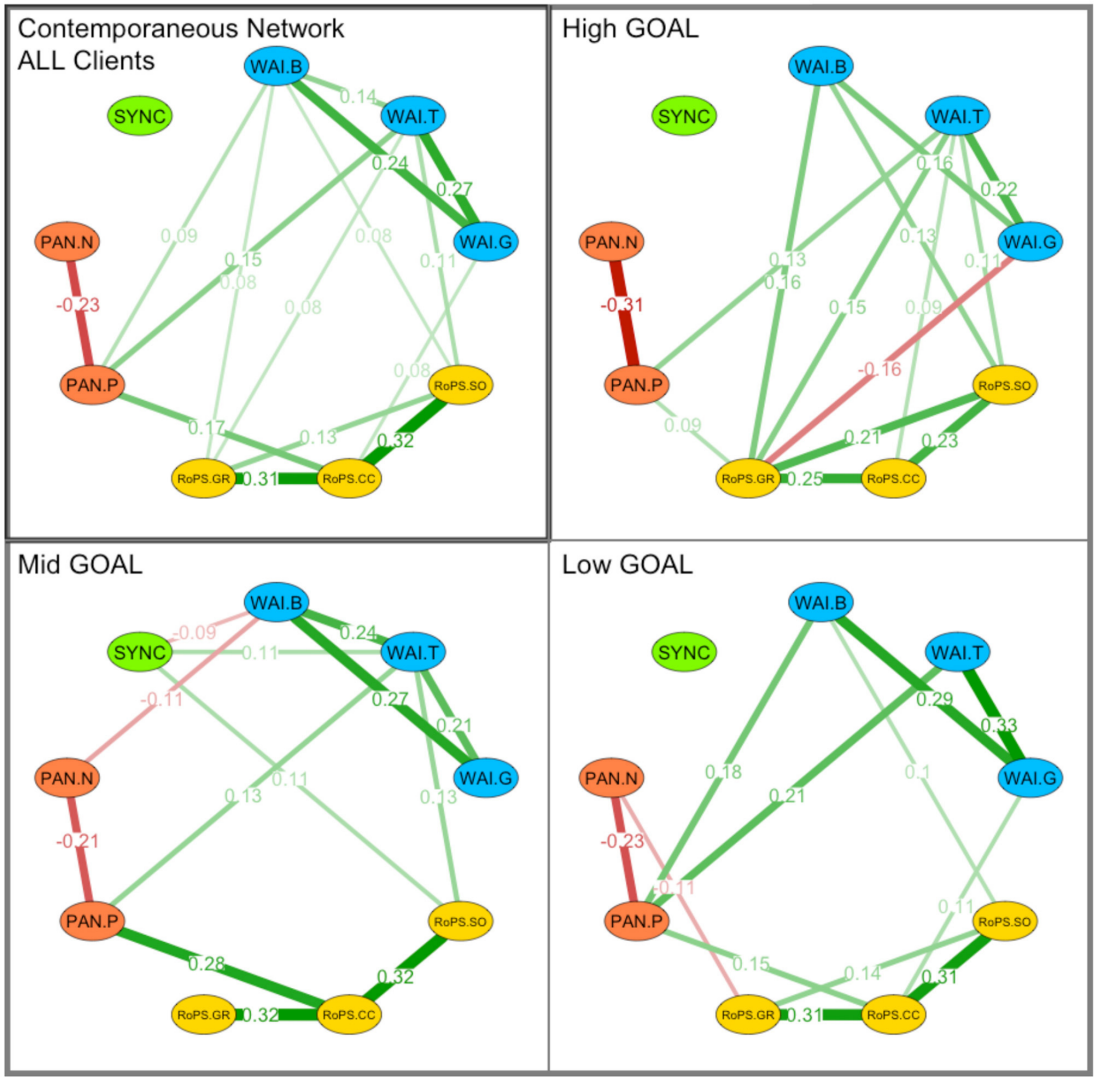
Contamporaneous network analysis by goal groups. Network analyses: Contamporaneous associations between synchrony (SYNC), working alliance (WAI), affective experience (PAN), and cognitive self-regulation (RoPS). Associations highlight nodes between the following variables: WAI.B, Working Alliance Bond; WAI.T, Working Alliance Task Setting; WAI.G, Working Alliance Goal Orientation; RoPS.SO, Result-oriented Problem and Self-Reflection.Self-Organization; RoPS.CC, Result-oriented Problem and Self-Reflection.Concrete Changes; RoPS:SO, Result-oriented Problem and Self-Reflection.Self-Organization; PAN.P, PANAS Positive Affect; PAN.N, PANAS Negative Affect; Associations are clustered by “ALL Clients” as well as groups of High GOAL attainment, Mid Goal attainment, and Low Goal attainment.

**Figure 11:**
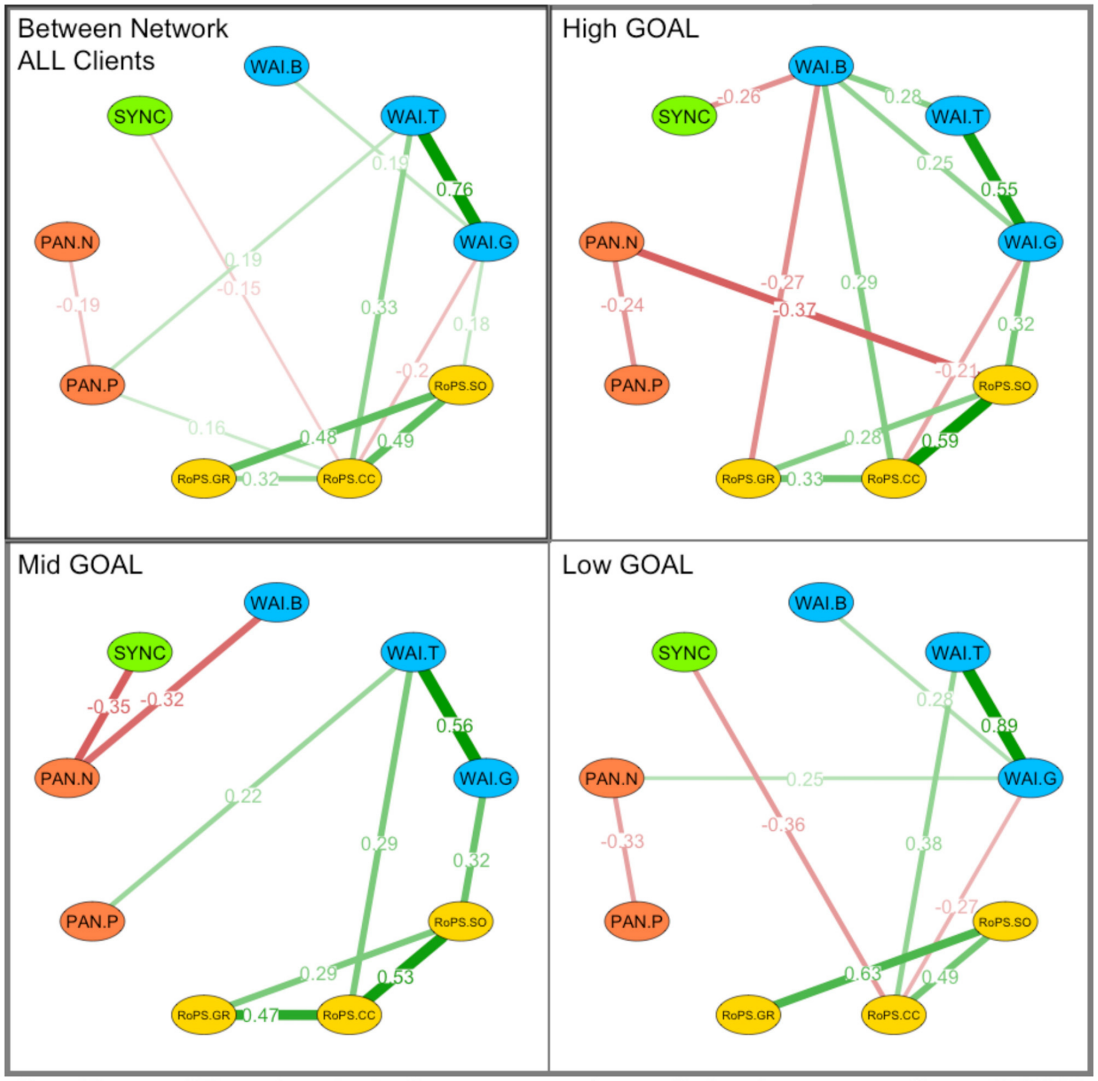
Between network analysis by goal groups. Network analyses: Between network analysis for associations between synchrony (SYNC), working alliance (WAI), affective experience (PAN), and cognitive self-regulation (RoPS). Associations highlight nodes between the following variables: WAI.B, Working Alliance Bond; WAI.T, Working Alliance Task Setting; WAI.G, Working Alliance Goal Orientation; RoPS.SO, Result-oriented Problem and Self-Reflection.Self-Organization; RoPS.CC, Result-oriented Problem and Self-Reflection.Concrete Changes; RoPS:SO, Result-oriented Problem and Self-Reflection.Self-Organization; PAN.P, PANAS Positive Affect; PAN.N, PANAS Negative Affect; Associations are clustered by “ALL Clients” as well as groups of High GOAL attainment, Mid Goal attainment, and Low Goal attainment.

In the contemporaneous network (associations between variables in the same session), synchrony was not associated with any other variable (Figure 10, Panel “ALL Clients”), but there are strong associations among the three process measures. The strongest contemporaneous association was found between positive affect and WAI-T, i.e., the high positive affect was associated with high-task orientation in the same session. The between-network analysis suggested that, in terms of average differences across dyads (i.e., on a group level), synchrony was negatively associated with concrete changes (RoPS-CC; *r* = −0.148; *p* = 0.046), such that less synchrony and more concrete changes were a common (significant) combination of this sample (Figure 11, Panel “ALL Clients”).

### Subgroups: Network Model Analysis

We then performed a larger number of these analyses in order to explore the networks of different subgroups: By dividing the sample into equally sized groups of *high outcome* (top 33%), *medium outcome* (middle 33%), and *low outcome* (low 33%) reported in the goal attainment scale, specific associations with synchrony were evident. We limit the description of these explorative assessments to synchrony, because this was our main variable of interest. Further details of the other associations may be found in the figures (Figures 9–11; Panels “High/Mid/Low GOAL”). In the *high-outcome* subsample (top 33% of goal attainment), synchrony had no significant association with any other variable in both temporal as well as contemporaneous networks (Table 4).

**Table 4:** Parameter estimates (and Standard Errors) for mixed effects models examining Hypotheses 1 and 3.

**Fixed effects**	**Hypothesis 1a (DV: PANAS-AB)**	**Hypothesis 1b (DV: RoPS-tot)**	**Hypothesis 3a (DV: RoPS-tot)**	**Hypothesis 3b (DV: RoPS-tot)**	**Hypothesis 3c (DV: RoPS-tot)**
Intercept	20.866^***^ (0.761)	3.145^***^ (0.039)	3.140^***^ (0.033)	3.130^***^ (0.025)	3.141^***^ (0.025)
Session	0.799^***^ (0.085)	0.070^***^ (0.004)	0.056^***^ (0.004)	0.050^***^ (0.004)	0.044^***^ (0.004)
PANAS-AB^†^			0.017^***^ (0.001)		0.011^***^ (0.001)
WAI-tot^†^				0.481^***^ (0.026)	0.395^***^ (0.027)
Synchrony	0.088 (12.733)	0.924 (0.617)	0.056 (0.004)	0.785 (0.545)	0.689 (0.527)
SYNC X PANAS-AB			0.70^t^ (0.042)		0.105^*^ (0.043)
SYNC X WAI-tot				−1.367^t^ (0.783)	−2.206^**^ (0.838)
ICC (Client)	0.570	0.497	0.446	0.447	0.422
ICC (Coach)	0.130	0.303	0.237	0.054	0.058
AICc	9233.258	1447.685	1276.623	1170.983	1092.471

t *p < 0.10;*

* *p < 0.05;*

** *p < 0.01;*

*** *p < 0.001*.

† *Process measures were centered at their grand mean*.

*DV, Dependent Variable; RoPS-tot, Overall Cognitive Self-Reflection; PANAS-AB, Affect-Balance; WAI-tot, Overall Working Alliance; SYNC, Non-verbal Synchrony; ICC, Intraclass Correlation; AICc, Corrected Akaike Information Criterion*.

This was not the case for the *mid-level outcome* group: For this subsample (mid 33% of goal attainment), numerous associations between synchrony and process variables were found in temporal and contemporaneous networks. Two positive associations were found in the contemporaneous network, namely, synchrony and WAI-B (*r* = 0.104; *p* = 0.030), and in the between-network analysis, namely, synchrony and WAI-G (*r* = 0.240; *p* = 0.033). This was again different in the *low-outcome* subsample: In the temporal network, synchrony was negatively associated with WAI-T (*r* = −0.088; *p* = 0.046) and in the between-network analysis, synchrony and RoPS-CC were negatively associated (*r* = −0.283; *p* = 0.038). The *mid-outcome* subsample provided the highest number of associations in the temporal network: Synchrony negatively predicted PAN-P (*r* = −0.119; *p* = 0.048), RoPS-GR (*r* = −0.127; *p* = 0.049), WAI-B/T/G (*r* = −0.097 to −0.166; *p* = 0.011–0.039), and it was negatively predicted by RoPS-SO (*r* = −0.125; *p* = 0.036). For the contemporaneous network, there was a negative association with WAI-B (*r* = −0.135; *p* = 0.027) and a positive association with RoPS-SO (*r* = 0.111; *p* = 0.028). The between-network analysis (Figure 11, Panel “Mid GOAL”) indicated that synchrony and PAN-N were negatively associated across dyads (*r* = −0.369; *p* = 0.001). Finally, the no-outcome group was very small (*n* = 8) and provided insufficiently dependable results that we do not report in detail here.

Considering the moderation effects reported for *Hypothesis 3*, the network analyses may be regarded as providing further evidence that nonverbal synchrony and other process variables are differentially associated in this sample of coaching clients. Most notably, we found out that, in highly successful dyads (*high GOAL* group), nonverbal synchrony plays a much less important role than in the other two groups (see models reported in Table 4).

## Discussion

The comprehensive data analysis exploring a multicultural sample of naturalistic coaching revealed a whole range of differential effects between process variables and outcomes. For reasons of conciseness, we discuss in detail two main results deemed relevant for the theoretical framework of the coaching literature on synchrony, self-regulation, and goal attainment. These hypotheses-driven results contain (I) the temporal nature of nonverbal synchrony in goal attainment and (II) the moderation effects of the working alliance in goal attainment. Generally speaking, it may be stated that process measures of coaching (applied weekly, after each session) indicated that a solid working alliance, a high level of goal reflection, and predominance of the positive mood predicted successful goal attainment.

### The Temporal Nature of Nonverbal Synchrony in Goal Attainment

Nonverbal synchrony was not associated with self-report measures (at the level of the same session) nor with global outcomes (on completion of coaching). Instead, multilevel- (Table 4) and temporal-network analyses (Table 3) uncovered that nonverbal synchrony and goal attainment were associated in a peculiar temporal manner: (a) synchrony showed a linear trend for a temporal decrease; (b) lower levels of synchrony in a previous session (*t–*1) predicted higher task orientation, higher goal setting, and higher goal reflection in the next session (*t*); and (c) in the group with average coaching success (mid 33%), nonverbal synchrony in a previous session predicted a lower level of bonding (WAI-B) in the next session. Similarly, nonverbal synchrony was also negatively associated with the level of bonding of the same session. These associations between nonverbal synchrony and bonding were not found in the less-successful group (low 33%), while, in the most successful group (top 33%), nonverbal synchrony was positively associated with bonding of the same session.

The above-mentioned phenomena (a) and (b) may be interpreted as indicative of a “correctional mechanism” that emerges at a point in time where the coaching process is perceived to be deteriorating. Higher levels of synchrony at the outset of the coaching process may indicate that greater effort is required in terms of nonverbally “getting onto the same page” or “attaining the same wavelength with each other,” which later becomes less important as coaching sessions progress successfully toward goal attainment. In contrast, in dyads where progress is perceived to “get off track,” as clients reported low cognitive and emotional self-regulation and low quality of coach–client relationship, higher levels of nonverbal synchrony may be interpreted as emerging efforts to correct the deteriorating quality of the coaching relationship or the yet unproductive coaching process. In other words, nonverbal synchrony could become more prominent and also more relevant in dyads where working alliance is not solid enough. Although counterintuitive at a first glance, this interpretation fits the finding that lower levels of synchrony in a previous session predicted higher levels of working alliance and cognitive self-regulation in the next session. A possible reason for this temporal association is our suggested function of nonverbal synchrony acting as a corrective mechanism in low working alliances. Our sample thus suggests that synchrony may primarily exist on a state level (Cohen et al., 2020; Zilcha-Mano, 2020). The temporal patterns of synchrony have been identified as a central factor in a recent model, aiming to integrate the mixed findings regarding the beneficial effects of synchrony on interpersonal outcomes (Mayo and Gordon, 2020). The authors point out that, in real life, individuals tend to move in and out of interpersonal synchrony, and that a continuous level of synchrony is clearly an exception in common social interaction. We think that this may also well be the case for the coaching data presented here.

Generally speaking, nonverbal synchrony could, therefore, be conceptualized as a way of being present with clients: It is the coaches' way of “*being with clients*” (Gendlin, 1969; Linder-Pelz and Hall, 2007; Silsbee, 2008; Divine, 2009; Sieler, 2010; Madison, 2012; Strozzi-Heckler, 2014) rather than their out-of-the-toolbox way of “*doing coaching*” session-by-session that is likely to make a significant difference in how clients feel capacitated to attain goals in coaching. All the more, as our temporal network analyses suggest that the creation of what we refer to as an “authentic environment” [i.e., the coach showing up authentically (Harter, 2002; Boucher, 2011; Sutton, 2020), which may invite a client to have the courage to do likewise] in coaching is of greater effect than movement coordination *per se*.

While previous coaching research, including nonverbal behavior in dyads (Schermuly et al., 2010; Ianiro et al., 2013, 2015; Ianiro and Kauffeld, 2014) showed that the working alliance depended on the degree of dominance and affiliation of coach–client interactions, our study focused on the moderating role of the working alliance in the association between nonverbal synchrony and self-regulation toward goal attainment. While these previous coaching studies acknowledge the aspect of reciprocity as a key element of interpersonal theory (Kiesler, 1996) and thus converge with the theoretical framework of interpersonal synchrony (Feldman, 2007, 2015), they differ in their focus on affiliation and dominance and how these interpersonal factors impact on the working alliance in coaching. As such, they complement the findings of the present study in how we can view nonverbal behavior as an interactional process that can be both the product of and a causal contributor to positive interactions as suggested in social psychology (e.g., Chartrand and van Baaren, 2009). In other words, nonverbal synchrony should not be interpreted in isolation of the context or from other variables such as goal attainment, working alliance, or affect.

Regarding the associations with goal attainment in (c) above, the negative relationship between nonverbal synchrony and bonding—notably in the group with average coaching success—was not anticipated in our hypotheses. It contrasts findings by Ramseyer and Tschacher (2011); Cohen et al. (2020); and Altmann et al. (2020). However, such an effect is not new in research on nonverbal synchrony and falls in line with other diverging outcomes on synchrony (Palumbo et al., 2017; Mayo and Gordon, 2020). A recent study interpreted a similar negative association to be a possible indicator for different aspects evident in idiographic vs. nomothetic samples (Ramseyer, 2020a). Indeed, a further study on nonverbal synchrony in psychotherapy pointed toward an optimal (middle) level of synchrony, where low nonverbal synchrony was found to be an indicator of dropout and high nonverbal synchrony to be a predictor of early termination (Paulick et al., 2018a). This also falls in line with recent work, where nonverbal synchrony in the third session of psychotherapy predicted lower success later in therapy (Lutz et al., 2020). We interpret the goal-related findings in the temporal networks (i.e., low synchrony being associated with high goal attainment while high synchrony being associated with low goal attainment and low goal orientation) as yet another indication that synchrony emerges as a correctional mechanism in dyads, and that we may have to look beyond average levels of synchrony toward more smaller-scale dynamics of synchrony, a phenomenon that has been called “symmetry building” and “symmetry breaking” (Boker and Rotondo, 2002). Our findings also indicate that high initial nonverbal synchrony not necessarily implies good contact between a coach and a client. This may be compared with findings in student dyads, where synchrony was higher in discussions of a conflictual type compared with discussions characterized by collaboration (Tschacher et al., 2014). The fact that, in the study investigating student dyads, the highest levels of synchrony were evident while students were engaged in a very specific “fun task” (building a menu of disliked foods) further suggests a dependency of the level of synchrony on the situation or the task at hand. In a similar vein, the success in collaborative tasks has been shown to be highest in weak coupling, i.e., not in totally synchronous behavior (Abney et al., 2015; Wiltshire et al., 2018). In the present study, the optimal level of nonverbal synchrony may not effectively only lie somewhere in-between too little (“bored-teenager effect”) and too much synchrony (“mime effect”) as described by Boker (2004) and illustrated in Ramseyer (2010). Instead, it may as well depend on the contextual situation and the characteristics of the verbal exchanges between interaction partners. As mentioned above, so-called “weak coupling” may, indeed, be an important condition for successful social or collaborative interaction (Wiltshire et al., 2018). Given the non-experimental character of the present study, this question remains unanswered, but we think that future studies should try to control for and specifically focus on contextual factors of coaching interactions (Erdoes et al., 2020).

### Moderation Effects of Working Alliance

Interaction effects in mixed model analyses showed that the effect of nonverbal synchrony on cognitive self-regulation (RoPS) largely depended on the expression of working alliance and mood. In dyads with high working alliance, nonverbal synchrony appears not to act as a beneficial factor for other process variables, while dyads with low working alliance showed a positive connection between synchrony and cognitive self-regulation (Figure 8B).

A different moderator pattern was found regarding affect balance: Dyads with high levels of affect balance were characterized by a positive association between synchrony and cognitive self-regulation (Figure 8A; Table 4). This further corroborates our claims that nonverbal synchrony needs to be considered in connection with other variables to make sense in coaching effectiveness. The moderator effect of mood came as a surprise, all the more as it was not hypothesized as part of the prediction model (Figure 1).

Generally speaking, the particular finding on the role of working alliance as a moderator suggested that coaching is a dynamic learning process with each coaching session forming more than the sum of its individual parts. Graßmann et al. (2020) report that working alliance was linked to but did not cause coaching outcomes. The findings in this study suggest that working alliance may be viewed to embody an interpersonal variable rather than an outcome variable. As such, it strengthens or weakens the direct relationship of nonverbal synchrony and self-regulation.

### Recommendations for Coaching Practice and Training

We recommend coaching providers to work with emotions, because they appear to strengthen clients' self-regulatory capacities and because coaches' moods and interpersonal behavior have been shown to impact clients' effectiveness (Ianiro and Kauffeld, 2014). Given that nonverbal synchrony may be viewed to partly work as a correctional mechanism, coaching training providers and practitioners may focus on honing their capacity to identify the quality of the coach–client relationship effectively at the outset of the coaching engagement. This may help to then be able to flexibly and spontaneously use nonverbal synchrony for the clients' effectiveness in coaching. Generally, we recommend coaches to be trained in being and staying spontaneous and flexible throughout their coaching engagements as it is not worthwhile starting to synchronize nonverbally in a linear manner. Other factors (i.e., task setting, bonding, affect balance) appear to be highly important too.

## Limitations

First, the coaching sessions analyzed in this study were not part of a randomized controlled trial; instead, they comprised a highly diverse “convenience sample” of naturalistic coaching sessions. However, the naturalistic character of our dataset may both be viewed as a limitation and an asset: The 184 dyads assessed across their individual processes allow insights into the dynamics that normally go unheeded in a traditional pre-to-post outcome study. Hence, we regard this dataset as an important—tentative—step toward more temporally oriented research on coaching. Second, with the exception of nonverbal synchrony, all post-session and post-coaching measures were based on the clients' self-reports, which poses the risk of potential bias in responses. Furthermore, the coaches' self-reports did not form part of the study design, which could have added the valuable perspective of coaches to our process analyses. Third, there was no control for initial psychopathology or other potentially influencing factors. Future studies should seek to more fully capture individual factors at the beginning of the coaching, capture both clients' and coaches' perspectives on a (wide) range of instruments, and assess outcomes on a broad range of factors. Long-term effects should be taken into consideration by follow-up measurements, capturing some of the dynamics that may unfold after the coaching has ended.

The operationalization of nonverbal synchrony based on frame-differencing methods comes with certain methodological restrictions already mentioned in the methods section. Most importantly, it should be restated that MEA captures the dynamics of movement, irrespective of the direction and quality (see Ramseyer, 2020b).

## Conclusion

This study provides evidence that coaching is not a linear input-output mechanism but a complex dynamic change-process (Erdoes et al., 2020). In particular, interaction terms in network models suggest that higher levels of synchrony may be interpreted as an indicator of some “correctional mechanism” that may emerge at a point in time where the coaching process is perceived to be deteriorating. Furthermore, the optimal level of nonverbal synchrony may highly depend on the contextual situation and the characteristics of the verbal exchange between a coach and a client. The current study enhances our understanding of the effects of the clients' self-regulatory and relational coaching processes in association with nonverbal synchrony as a yet not sufficiently explored phenomenon. We hope that the complex interactions reported here could narrow the gap regarding our understanding about how coaching as a process works to produce change in and for clients. This study answers calls from coaching scholars (Myers, 2017) to identify a direction for future research on the coaching process rather than specific techniques associated with any particular method.

## Data Availability Statement

The data supporting the conclusions of this article will be made available by the authors, on reasonable request without undue reservation.

## Ethics Statement

The studies involving human participants were reviewed and approved by FEWEB Research Ethics Review Board/Vrije Universiteit Amsterdam, Department: Management & Organization. The patients/participants provided their written informed consent to participate in this study.

## Author Contributions

TE as the main author is the Ph.D. researcher. She conducted the research project on her own, supervised by Dr. Prof Erik de Haan at Vrije Universiteit Amsterdam, The Netherlands. TE conceptualized the introduction, the methodology, conclusion, parts of the discussion, and the limitations sections, She used the MEA (motion energy analysis) software developed by FR. Her IT support prepared the data for analysis and conducted the assessment of movement. FR conducted the complex data analysis based on the lines of hypotheses provided by TE. Both authors contributed to the final version of this article.

## Conflict of Interest

The authors declare that the research was conducted in the absence of any commercial or financial relationships that could be construed as a potential conflict of interest.
